# Achieving a Biocircular Economy in the Aquaculture Sector Through Waste Valorization

**DOI:** 10.3390/toxics13020131

**Published:** 2025-02-11

**Authors:** Setyo Budi Kurniawan, Azmi Ahmad, Muhammad Fauzul Imron, Siti Rozaimah Sheikh Abdullah, Ahmad Razi Othman, Hassimi Abu Hasan

**Affiliations:** 1 Department of Chemical and Process Engineering, Faculty of Engineering and Built Environment, Universiti Kebangsaan Malaysia, UKM Bangi 43600, Selangor, Malaysia; setyobk@ukm.my (S.B.K.); rozaimah@ukm.edu.my (S.R.S.A.); ahmadrazi@ukm.edu.my (A.R.O.); hassimi@ukm.edu.my (H.A.H.); 2 Research Center for Environment and Clean Technology, National Research and Innovation Agency (BRIN), Jakarta Pusat 10340, Indonesia; 3 Department of Petrochemical Engineering, Politeknik Tun Syed Nasir Syed Ismail, Pagoh 84600, Johor, Malaysia; azmi.ahmad@ptsn.edu.my; 4 Study Program of Environmental Engineering, Department of Biology, Faculty of Science and Technology, Universitas Airlangga, Campus C UNAIR, Jalan Mulyorejo, Surabaya 60115, Indonesia; 5 Sanitary Engineering Section, Department of Water Management, Faculty of Civil Engineering and Geosciences, Delft University of Technology, Stevinweg 1, 2628 CN Delft, The Netherlands; 6 Research Centre for Sustainable Process Technology (CESPRO), Faculty of Engineering and Built Environment, Universiti Kebangsaan Malaysia, UKM Bangi 43600, Selangor, Malaysia

**Keywords:** aquaculture wastewater, environmental pollution, sludge, nutrient, biofertilizer, biocircular economy

## Abstract

Aquaculture wastewater treatment not only assists in alleviating the scarcity of clean water for daily usage and environmental pollution, but also generates valuable byproducts. This paper aims to review the generation of wastewater from the aquaculture sector, its characteristics, and available treatment technologies, while comprehensively discussing the adoption of a biocircular economy approach through waste valorization. With rich nutrients, such as nitrogenous compounds, and the presence of phosphorus in the aquaculture effluent, these aspects could be explored and valorized into biofertilizers, broadening their application in aquaponics and hydroponics, as well as in algae and daphnid cultivation. Biofertilizer can also be used in agriculture because it contains essential elements needed by plants. Thus, methods of converting nutrients into biofertilizers in terms of sludge recovery can be accomplished via anaerobic and aerobic digestion, drying, composting, and vermicomposting. Moving forward, aquaculture effluent recovery is addressed under the biocircular economy by re-engaging aquaculture wastewater effluents into the production cycle. The enhancement of aquaculture effluents and biomass for uses such as aquaponics, hydroponics, algae cultivation, daphnid co-cultivation, and biofertilizers presents valuable opportunities for nutrient recovery while ensuring that non-toxic wastewater can be safely discharged into external water bodies. This approach has the potential to revolutionize wastewater treatment in aquaculture, shifting the economic model of wastewater management from a linear system to a circular, more sustainable one.

## 1. Introduction

Aquaculture production has become a fast-growing sector in the last decade in order to meet the global demand for supplying animal protein. However, because this sector consumes high volumes of water, aquaculture production could alter the water characteristics of biological and chemical components, eventually generating large amounts of effluents resulting from the biological activities of the cultured species, especially during the selection of final products [1]. Because the aquaculture effluent is rich with nutrients, typically due to the residue feed and metabolic waste, their release leads to implied direct, negative effects on the environment [2,3,4]. Aquaculture production generates raw sludge consisting of more than 90% water and rich nutrients of organic solids, for example, nitrogen (N) and phosphorus (P) (in most cultivation systems, especially ponds, flow-through, tanks, and RASs) [5]; this must then be treated and re-used [6] to remove pathogens (including bacterial, fungal, algal, and parasitic pathogens), increase dewaterability, and control malodor for safe disposal and space-saving [7]. In addition, the phosphate rock reserve that has been used to manufacture P fertilizer is estimated to be exhausted within 50–300 years [8], thus raising a critical issue for the future supply of P fertilizer. N-based fertilizer production through the Haber–Bosch process produces 1.6 tons of carbon dioxide, which poses a threat to the global goal of decarbonization [8]. With the presence of N and P in sludge, recovering those nutrients is vital for crop growth. Various methods have been proposed to convert effluents into useful biofertilizers by using aerobic and anaerobic digestion [9,10], drying [11], composting [12], and vermicomposting [13]. In turn, the recovered nutrients can be used to produce biofertilizer, reduce or eliminate water and soil pollution, as well diminish the operation cost for the sludge treatment [14]. This concept is known as the “circular economy”.

The concept of the “circular economy” is based on a few principles, such as “cradle to cradle”, looped and performance economy, industrial ecology, blue economy, regenerative design, and biomimicry, all of which emerged as alternatives to the traditional “take–make–dispose” (linear) economic model [15]. By design, the biocircular economy could convert wasteful effluents into valuable products and transform them into more environmentally friendly fertilizers [16]. These fertilizers can then become substitutes for the current conventional fertilizers, whose sources are almost depleted.

The concept of circular economy in the aquaculture sector is defined as an integration of farming technologies, waste treatment, and waste valorization [17,18]. This approach is designed to optimize the production of aquaculture commodities, reduce generated waste/wastewater, reduce its potential effect on the environment, recycle valuable waste/wastewater, and utilize technology to convert waste into valuable materials to achieve sustainable production [19,20]. Recent updates mention the focus on the biocircular economy approach for the aquaculture sector. The biocircular economy in aquaculture is the incorporation of biomass byproducts and biotechnologies to achieve sustainability [1]. Algal technologies (such as wastewater treatments or co-cultivation strategies) were mentioned by previous researchers as one of the most feasible methods to achieve a biocircular economy [21,22]. A recent article elaborated on the integration of wastewater treatment, biomass valorization, and biotechnological approaches in aquaculture to achieve a biocircular economy [23]. However, the discussion detailing and emphasizing solid waste (sludge) in aquaculture is still very limited. In addition, approaches to the utilization of natural coagulants/flocculants to produce metal-free sludge and the valorization of aquaculture wastewater into daphnids are currently limited.

To fill these gaps, this article systemically reviews aquaculture wastewater generation, the characteristics of its effluents (wastewater and sludge), and a comparison of available treatment technologies for aquaculture wastewater. This review also describes the valorization of aquaculture effluents, including their potential use as biofertilizers for algae and daphnid cultivation, and their integration with the hydroponic system. This article emphasizes the framework of the biocircular economy and how to achieve it in the aquaculture sector. The findings and concepts presented in this article are expected to contribute to the perspective of the biocircular economy, especially in the aquaculture sector, as well as provide insights into the practice of the biocircular economy approach.

This review paper was structured following the PRISMA method. Source extraction was conducted from the SCOPUS database (years 2005 to 2025) using the following keywords: “aquaculture”, “biocircular economy”, “biofertilizer”, “circular economy”, “cleaner production”, “effluent”, “fertilizer”, “nutrient”, “recovery”, “technology”, “treatment”, and “wastewater”. Initial extraction resulted in more than 1500 articles matched with the keywords. The number of articles was then narrowed to 174 papers, following the further refinement criteria of (i) studies that should focus on aquaculture/circular economy/wastewater treatment, (ii) a minimum of two aforementioned keywords appearing in the paper, (iii) the study focusing on the nutrient/resource recovery, and (iv) English being the main language used. The discussion was synthesized by extracting and describing important information contained in the selected sources, which were then summarized as figures, tables, and descriptive paragraphs [24].

## 2. Aquaculture Activities and Wastewater Generation

### 2.1. Aquaculture Activities

**Figure 1:**
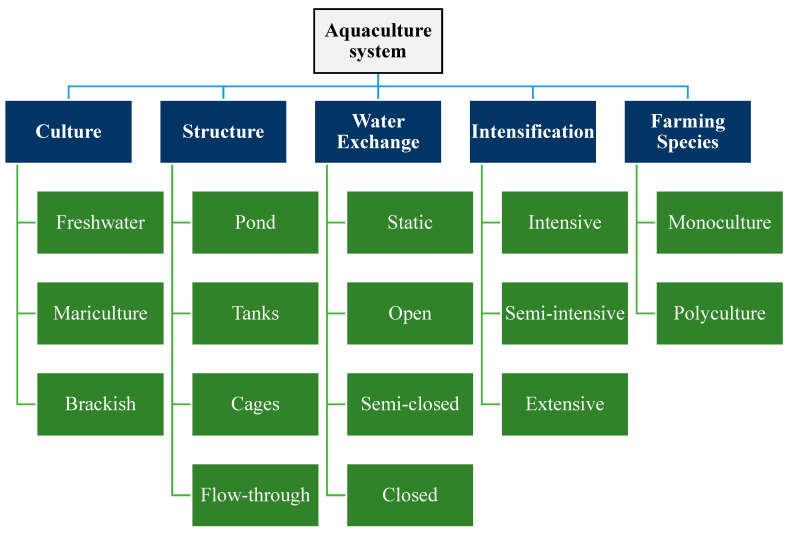
Classification of aquaculture systems.

Aquaculture is the farming of aquatic species commonly associated with fish [25]. However, it covers animal- and plant-based aquatic species, such as fish, crustaceans, seaweeds, algae, microalgae, and mollusks. The sector also involves farming or culture in freshwater, brackish, and seawater (mariculture), depending on the type of aquatic species. The classification of aquaculture systems based on their culture, structure, water exchange, intensification, and farming species are depicted in Figure 1.

#### 2.1.1. Classification Based on the Culture System

Based on the culture, aquaculture is divided into three main categories, which are freshwater, mariculture, and brackish water culture. Freshwater aquaculture primarily focuses on the cultivation of fish, as well as other species such as crab, shrimp, and aquatic plants. This is achieved via the utilization of various culture systems, including ponds, flow-through systems, recirculation aquaculture systems (RAS), and other inland waterways [26]. According to previous reports, freshwater aquaculture accounted for 64% of the worldwide fish farming production in 2016, including species such as carp, tilapia, and crustaceans that thrive in freshwater environments [27,28]. However, in 2018, this proportion experienced a slight decline, settling at 62.5%. The persistence and quality of freshwater aquatics are heavily dependent on climatic and hydrologic regimes [29]. The practice of mariculture, also known as marine culture, is conducted either in the open sea or along the coastal regions, with the specific classification of water types determined by the concentration of salinity. As stated by Ahmed and Thompson [30], freshwater, brackish water, and seawater may be distinguished based on salinity levels of <0.05 ppt, 0.5–30 ppt, and >30 ppt, respectively. Mari- and brackish water cultures involve the utilization of artificial structures, such as cages or ponds, for the purpose of cultivating fish. At present, around 33% of aquaculture production is conducted in marine environments, with this sector exhibiting a notable expansion trend that involves progressively venturing into deeper oceanic regions [31]. In the year 2018, the worldwide output of mariculture amounted to a total of 30.8 million tons. Out of this, 37.5% was attributed to the global aquaculture industry, while the continent of Asia alone accounted for a significant proportion of 88.69%, equivalent to 72.8 million tons. The primary species consisted predominantly of economically valuable fish, including salmon, seabass, seabream, barramundi, and trout, alongside bivalve mollusks and seaweed [30].

#### 2.1.2. Classification Based on the Structure

Pond culture is a widely utilized approach in aquaculture production, which can be categorized into three distinct categories, namely levee ponds, watershed ponds, and depression ponds, depending on the source of water supply [32]. Ponds can keep a volume of water within a designated region by the presence of a water intake, subsequently facilitating its outflow. Aquatic systems that exhibit static characteristics are not dependent on water exchanges for the maintenance of their water quality. This is due to the presence of internal natural processes that effectively cleanse the water. In the aquaculture system, the introduction of fresh water is employed as a means to replace the contaminated water following two or more cycles of fish harvesting. The rate of fish production in this system is contingent upon the daily quantity of feed input while simultaneously ensuring the maintenance of water quality [33]. Besides the pond system, tanks are also used in freshwater culture. Aquaculture tanks serve as containers utilized in the practice of cultivating aquatic creatures, including fish, shrimp, and algae [34]. The tanks have been purposefully engineered to ensure the maintenance of ideal water quality, temperature, and oxygen levels, hence facilitating the achievement of effective aquaculture production [35,36]. Aquaculture tanks are considered indispensable for the effective cultivation of diverse aquatic creatures and significantly contribute to the worldwide aquaculture sector.

Cage culture, sometimes referred to as net-pen culture, involves the utilization of a suspended net structure inside the water column, encompassed by a flotation system surrounding its periphery [37]. Typically, the net is suspended in a geometric arrangement, commonly square or rectangular, consisting of four sides and a bottom. However, certain cage systems utilize circular nets as an alternative layout. The dimensions of a cage might exhibit significant variation contingent upon the requirements of the culturist [38]. A diminutive cage may encompass only a few square meters, while more expansive cages, specifically designed for use in offshore regions, can span an area of up to 500 m^2^. The range of depths for the cages typically span from 3 to 15 m. The enclosure is secured in place by one or many anchor lines that extend outward from its perimeter. In the majority of instances, a farm consists of several cages that are either closely positioned or physically linked together to create a substantial array [39]. In the United States, marine cage systems often utilized for salmonids normally have a collective arrangement of 10 to 50 cages, which are securely anchored together inside a singular expansive array. The output of salmonids at the majority of commercial plants is projected to reach a minimum of 25 metric tons per year. Marine farms often exhibit higher production levels, with the biggest facilities potentially surpassing an annual output of 500 tons. Marine cage systems account for over 90% of the global production of farmed salmonids.

Flow-through systems are commonly employed for salmonids and consist of a sequence of raceways or tanks through which water flows, ultimately being discharged into an external water body. The utilization of these systems is facilitated by the presence of a sufficient uncontaminated water supply, and it is recommended that they be positioned at a downstream location relative to a diverted river or stream. In a study conducted by Ngo et al. [25], the authors provided estimates for the hydraulic retention time necessary for the discharge of water in both single raceways and series of raceways. The estimated values were around 300–420 m^3^/kg and 66 m^3^/kg, respectively, with a time frame of 1 h.

#### 2.1.3. Classification Based on the Water Exchange

A significant portion of worldwide aquaculture production relies on conventional pond culture techniques. The ponds under consideration have a static nature, wherein there is an absence of water exchange throughout the cultivation season [40]. Further replenishment may be necessary in order to compensate for the process of evaporation. Extensive static pond culture is commonly employed due to significant challenges associated with preserving water quality in the presence of a substantial biomass of cultured fish inside a given volume of static water [41].

An open system refers to the practice of utilizing the surrounding environment, such as cages [37], as a means of cultivating organisms. In this system, the cultured organisms are contained or safeguarded within the farm, which is situated in a large body of water, such as a lake or an ocean. The purpose of this arrangement is to ensure that the quality of water is upheld through natural flows and processes. The system does not include any artificial circulation of water either through or within it [42]. The cage system is categorized as an open system when it is situated in a vast aquatic environment, such as an ocean or an estuary. The maintenance of water quality is facilitated by natural currents and tides. The presence of seasonal fluctuations in the environment gives rise to substantial variations in growth rates, which is a significant drawback of open systems.

The term “semi-closed aquaculture” pertains to the cultivation of a particular species in a land-based setting, where there is a controlled exchange of water between the farm and a nearby natural river [43]. The discharge of wastewater from the ponds into the nearby river occurs simultaneously with the replenishment of the farm by the pumping of new water back into the system (e.g., raceways) [44]. Closed-system aquaculture involves the cultivation of aquatic species in controlled environments, such as RAS [45], situated on the land. The implementation of recirculation technology involves the utilization of filtering technologies to cycle water and subsequently reintroduce it into the aquaculture system. This procedure facilitates the preservation of water quality while minimizing the interaction with natural water bodies. Closed-system aquaculture is often regarded as a very ecologically sustainable approach for the cultivation of aquatic species [46].

#### 2.1.4. Classification Based on the Intensification

The measure of culture intensity refers to the density of aquatic organisms within a certain area or volume, as well as their capacity for natural productivity. This concept is commonly categorized into three systems: intense, semi-intensive, and extensive [41,47,48]. Intensive aquaculture systems need substantial stocking densities and feed inputs to optimize productivity since the fish are provided with significant quantities of protein-rich feed [47,49]. The energy losses associated with feed intake are predicted to be minimal, and the waste discharges are indirectly released into external water sources. Extensive agricultural systems have reduced levels of human intervention, leading to decreased yield and a lower stocking density, estimated at 500 kg per hectare. Semi-intensive systems lack a definitive criterion for differentiating between intense and extensive systems. The organisms in question depend on a naturally occurring food source, which is supplemented or enhanced by fertilization while also accommodating a higher density of organisms inside the culture system.

#### 2.1.5. Classification Based on the Farming Species

Polyculture and monoculture are frequently classified as fish farming techniques involving the cultivation of a single species or many species during a specified culture time. While monoculture is the prevailing method employed in RAS, the potential adoption of polyculture, which involves the production of many species, holds promise for addressing some constraints associated with this particular production system [50]. The efficacy and sustainability of production systems may be enhanced by the use of polyculture, as evidenced by empirical research. The use of polyculture can enhance system functionality through the exploitation of cohabitation and interactions among diverse species [51,52,53]. This approach facilitates improved usage of feed resources, minimizes feed losses, reduces waste through recycling, and optimizes the utilization of culture space. According to previous studies conducted by Dickson et al. [47] and El-Sayed [26], it has been shown that polyculture exhibits superior efficiency in terms of food use for fish production when compared to monoculture.

### 2.2. Waste Generation from Aquaculture

**Figure 2:**
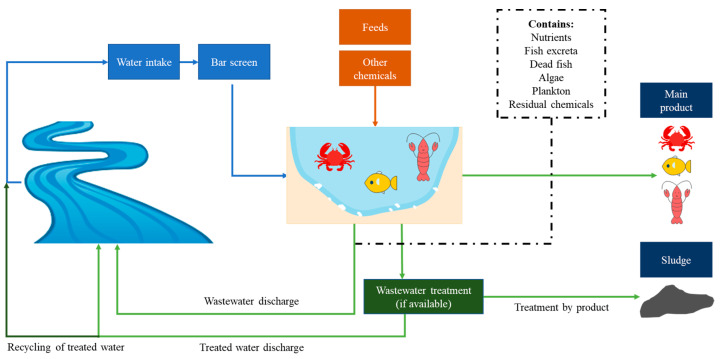
General process of aquaculture production.

A simple diagram of aquaculture production, referring to pond systems, is depicted in Figure 2.

Aquaculture production involves three common stages: breeding, rearing (grow-out), and harvesting. Feeding is the main element that can be acquired from plant- and animal-based processed (undergoing the process of drying, mixing, and fermenting pelleting) and non-processed feeds (live, fresh, and frozen), and the stage is very expensive because it covers almost 60% of the overall production cost [54,55]. Various chemicals, such as antibiotics, disinfectants, anesthetics, fertilizers, and antifoulants, are used to ensure aquaculture production is profitable and successful [56,57]. However, the use of these chemicals is generally not governed by specific control legislation or risk assessment. Nevertheless, certain countries and international agencies have implemented regulations and guidelines to ensure these chemicals can be controlled and not excessively used to avoid harm to cultured species [58]. Some residues and or intermediates of the said chemicals were found in wastewater discharged from aquaculture systems [55,59,60]. The effluents from the feed residues, feces, and dead cultured species, along with the residual chemicals used, may considerably pollute the waters [61].

When cultured species reach the maturity period, depending on the species type, harvesting takes place by slowly draining out the water from the culture, thereby ensuring the cultured species are in good condition [55]. Aquaculture wastewater is generated during the harvesting stage for flow-through and raceway ponds, as well as during farming in recirculation and intensive farming systems. The drained water (including sludge) discharged to external water bodies (rivers or lakes) is considered waste from aquaculture. Globally, more than 80% of the wastewater is discharged to surrounding water bodies untreated [62]. For the aquaculture sector, an estimated 20.15 m^3^/kg production/year of wastewater is generated, with the average data taken from multiple culture systems, i.e., intensive, extensive, and flow-through [63].

This wastewater generation and its influence on surrounding bodies of water can be reduced, firstly, by applying simple, low-cost treatment processes, such as coagulation–flocculation [64,65], to treat the wastewater into an acceptable range of certain parameters (as per locally established regulations and guidelines). Secondly, instead of using common culture systems like ponds, replacing the current method with the more efficient, cleaner technology of recirculating aquaculture system (RAS) [66] by treating the wastewater and recycling it back to the culture system for reuse is encouraged [36,67]. The coagulation–flocculation technique using alum removed up to 92% of turbidity, 94% of TSS, 39% of TN, and 84% of TP [68], while the utilization of green coagulants such as chitosan and *Moringa oleifera* seeds were reported as alternative options [64,69]. Depending on the integrated treatment, RAS reduced up to 71% of TSS, 95% of TN, and 85% of TP [70]. RAS outperformed conventional wastewater treatment systems because it can reduce wastewater generation by >90% [71], because the conventional system did not contribute to any wastewater generation reduction. However, the use of sophisticated technology and capital investment of RAS might be challenges to reconsider the use of this system [71,72].

## 3. Characteristics of Aquaculture Effluents

**Figure 3:**
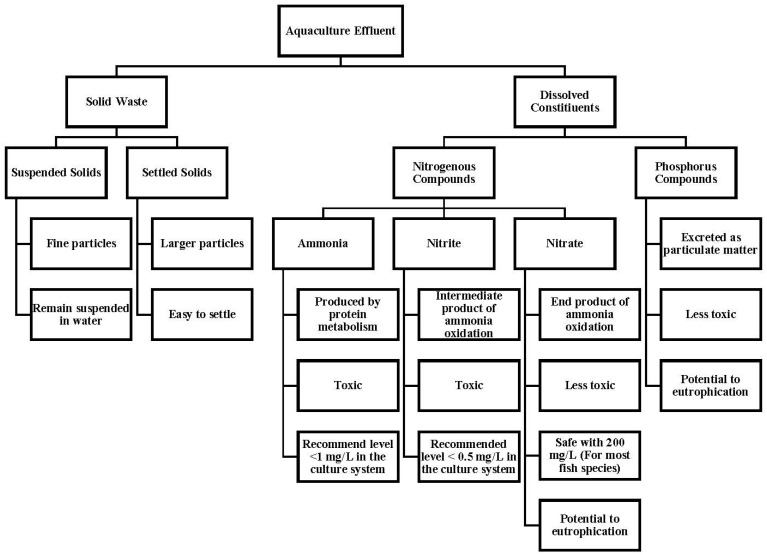
Simplification of the constituents and characteristics of aquaculture wastewater effluents.

Concerns about the environmental effects of water pollution from aquaculture wastewater effluents have been discussed over the years. Organic contents from fish feed and feces may cause environmental deterioration in external water bodies [73]. The characteristics of aquaculture effluent are highly varied, depending on the system used and the cultured species [46,74,75]. Generally, aquaculture effluents can be classified into two segments, namely solid waste and dissolved waste [56,76], as shown in Figure 3.

### 3.1. Solid Waste

Solid waste can be derived from uneaten feed or the excreta of cultured fishes. Suspended solids from these effluents are difficult to remove, and require proper treatment, such as coagulation or sedimentation, due to their fine particle attributes; in comparison, settled solids can be easily removed in less time [55,76]. The presence of solid waste in the culture system, especially large-settled particles, could clog fish gills, leading to death. Furthermore, the longer solid wastes remain in the culture system, the greater the likelihood of increased aerobic bacterial activity. In turn, this leads to increased chemical oxygen demand (COD) and biochemical oxygen demand (BOD), which diminish the levels of dissolved oxygen in the culture system [77].

Sludge is a common solid waste that is also known as sediment, ooze, pond bottom soil, or mud; it is originally suspended solids and later deposited at the bottom of a culture system once it settles down [78]. Suspended solids are considered the most dangerous constituents in the aquaculture system if not managed or treated properly [77]. The amount of sludge retained in a culture system or released into external water bodies depends on several factors, such as the cultured species, feed supply, and effective feeding [78,79,80]. If managed properly, sludge should not be regarded as waste, due to its valuable contents, including nutrients, energy, and large amounts of water. Nutrients from sludge are essential elements for plant growth because they can be reproduced as biofertilizers. Sludge can be recovered depending on the culture system introduced in the aquaculture sector, such as pond, raceway/flow-through, and RAS.

Because the pond is the most common and traditional method of culture, no special water treatment is involved; instead, it only relies on the internal process wherein solid wastes settle down and accumulate over a certain period to become sludge [76]. The sludge can then be cleaned after certain harvesting cycles using desilting. The raceway system, which requires a high level of water exchange, can discharge solid wastes from the culture system [76]. The raceway provides less than an hour of retention time, and can collect solid wastes in sluggish areas into the offline basin [76]. In comparison, RAS is better at treating aquaculture wastewater, and can remove larger solid wastes through sedimentation, whereas suspended solids (fine particles) can be removed via additional screen filters via sedimentation [76]. RAS is expected to reduce organic matters and suspended solids by around 85–95%, as well as 65–96% of P after treatment [76,81].

### 3.2. Dissolved Wastes

Dissolved solids, which are contributed by nitrogenous (ammonia, nitrite, and nitrate) and P compounds, are constituted from protein and originate from feed. An estimated 25–50% protein can be taken by the cultured species, and the rest are not retained in their bodies; thus, the residues are released into the culture system (water body) and pollute the waters. Ammonia is the final product of protein metabolism [82], and an increase in ammonia can trigger stress among the cultured species and increase the likelihood of disease infection [78,83]. The other effects of ammonia increase include the accumulation of ammonia in blood and tissue of the cultured species, the reduction of oxygen consumption, and negative effects on metabolic enzyme activity [78].

Nitrite is a transitional compound resulting from ammonia oxidation to nitrate, and is mainly characterized by instability and toxicity. A high concentration of nitrite in a water body can affect the oxygen-carrying capacity of the cultured species, thus causing an anemic state that can lead to mortality [78]. The final component of ammonia oxidation is nitrate, which is less toxic than ammonia and nitrite. A study by Learmonth and Carvalho [75] indicated nitrate concentration as high as 200 mg/L is still considered safe because it neither changes the water quality nor disturbs the cultured species, including aquatic plants. However, nitrate concentration may increase over time via accumulation. Thus, along with additional factors, such as the frequency of water exchange in the culture system, increased nitrate concentration could lead to pollution in the external water bodies [84,85].

The feed is an important component that ensures the proper growth of the cultured species, which may later contribute to the P content in wastewater as the result of the decomposition of excess feeds. The feed exists in inorganic and organic forms, and it is excreted from the cultured species, while the residues of the uneaten feed contribute to soluble P [86]. An estimated 80% of the P existing in a culture system is discharged to the external water body [87,88], and most of the P (in fecal and particulate P) accumulates into sludge after settling [88]. Releasing P into the external water bodies changes the P dynamic which, in turn, could promote algal growth and accelerate eutrophication with the presence of high P. Eutrophication is also considered a serious threat to the water quality because it decreases the biodiversity and function of other aquatic ecosystems with the presence of excessive growth of algae and its accumulation in response to the increase in nutrient inputs [89]. Examples of the aquaculture effluent characteristics of the retained nutrients are presented in Table 1.

Table 1 shows that nitrogenous and phosphate compounds exist in aquaculture wastewater, and the major percentage is contributed by nitrate. Although nitrate is less toxic than nitrite or ammonia, it can cause chronic effects on some aquatic organisms due to the interaction with other environmental stressors [90,91]. In RAS, nitrogenous compounds are relatively higher than in raceway/common pond systems. RAS is used to minimize water input by reusing treated water as well as improving waste management [92]. However, the continuous water recirculation in the system causes ammonia accumulation as the result of fish metabolism of protein deamination from residual feeds [92]. Nitrification in the system may also cause the accumulation of toxic nitrite because denitrification rarely occurs in an RAS [93]. Table 1 indicates the possible pollution of water bodies caused by the high nutrient content in aquaculture wastewater. Furthermore, proper wastewater treatment is required to recover those rich nutrients and convert them into potential biofertilizers.

**Table 1:** Characteristics of nitrogenous and phosphorus compounds in aquaculture wastewater effluents.

Country	Culture System	Species	Retained Nutrients from Aquaculture Wastewater (mg/L)							Reference
			TN	NH_3_	NH_4_^+^	NO_2_^−^	NO_3_^−^	TP	PO_4_^3−^	
China	Pond	Yellow catfish	3.60	-	2.35	0.134	0.51	0.23	-	[94]
China	Pond	Carp	-	-	0.58	0.04	-	-	-	[95]
Canada	RAS	Eel	-	1.6	1.6	0.05	17	-	3.6	[96]
Ireland	Raceway/RAS	Perch	-	-	0.013–193.0	0.007–3.28	0.4–110.7	-	0.007–92.0	[3]
Mexico	Pond	-	-	0.12	4.8	0.02	5.1	-	11.9	[97]
Netherlands	-	Turbot	41.3	-	0.48	0.146	40.7	4.96	-	[98]
Norway	RAS	Salmon	-	-	-	-	18.1	2.5	-	[50]
Malaysia	Pond	Catfish	-	24.5	-	-	11.9	-	0.07	[99]
South Africa	RAS	Tilapia	-	-	4.6	-	140	-	15	[100]
Switzerland	RAS/hydroponic	Nile tilapia	-	-	≤0.1	0.3	152.8	-	16.1	[101]
Saudi Arabia	-	Shrimp	-	-	443	28.7	125.5	-	5.8	[102]

TN = total nitrogen, NH_3_ = ammonia, NH_4_^+^ = ammonium, NO_2_^−^ = nitrite, NO_3_^−^ = nitrate, TP = total phosphorus, PO_4_^3−^ = phosphate.

## 4. Bioeconomy and Available Wastewater Treatment Technologies for the Aquaculture Sector

**Figure 4:**
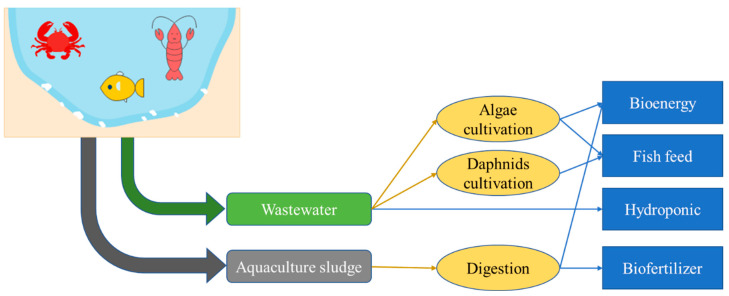
Valorization of aquaculture effluents and biomass by biotechnology.

“Bioeconomy”, “bio-based economy”, and “biotechonomy” are similarly defined as economic activities in a particular sector that involve the utilization of biomass and/or biotechnology to produce services/products/energy [103]. Bioeconomy refers to the efforts exerted to achieve sustainable production and consumption involving recent technological advances with fewer byproducts and/or chemical residues [104]. Bioeconomy is closely related to effluent valorization, which creates valuable products in some industrial sectors [105,106,107]. In the aquaculture sector, several studies on the valorization of generated effluents have been conducted [108,109], as inland aquaculture systems account for 62.5% of total farmed fish production [110]. The valorization of aquaculture effluents and biomass can be achieved using several methods, as summarized in Figure 4.

The focus of aquaculture wastewater valorization is to convert its pollutant parameters (mostly nutrients), which may produce side products that have additional value. The conversion of nutrient contents in aquaculture wastewater can be conducted in three main products: daphnids, algae, or plants. Different cultivation systems can be selected based on the characteristics of the wastewater being processed. Very high concentrations of nutrients characteristics are suitable for algae cultivation and hydroponic scheme [111], whereas moderate concentrations of nutrients are more suitable for daphnid cultivation [112]. For aquaculture sludge, valorization is focused on concentrating and converting the nutrient contents into liquid form. Although the direct utilization of aquaculture sludge has a positive effect on plant growth, it produces an unpleasant odor caused by the degradation of organic materials and N in the sludge [113]. Related to this, the valorization of aquaculture wastewater and sludge places additional value on the previously unwanted waste because they can make contributions by generating additional revenue and reducing the cost of running aquaculture production systems [114,115].

The valorization of aquaculture waste is an essential component of waste treatment technologies. The selection of treatment technologies can affect the effluent characteristics and byproducts [106]. Several options are available for the treatment of aquaculture wastewater before discharging it into surface water, including physical, biological, physicochemical, and hybrid processes, as previously detailed by Ahmad et al. [116]. Various technologies may then be used to remove pollutants, mainly consisting of suspended solids, nutrients, and organic matter (as tabulated in Table 2).

**Table 2:** Commonly used technologies for aquaculture wastewater treatment.

Type of Treatment	Treatment Unit	Operational Conditions	Removal	Reference
Physical	Filter	Media: *Crassostrea rhizophorae* and *Crassostrea gigas* HRT: 6 h Volume: 50 L	Turbidity: 62.1% TSS: 70.6% TVS: 36.1% BOD: 17.5%	[117]
Physical	Filter	Media: Sand and anthracite HRT: 80 min Filtration rate: 12 m^3^/h	Turbidity: 92%	[118]
Physical	Filter	Media: *Saccostrea commercialis* HRT: 24 h Volume: 10 L	TSS: 12% TN: 28% TP: 14% NH_4_^+^: 76% NO_3_^−^: 30% PO_4_^3−^: 35%	[51]
Physical	Membrane filter	Length: 60 mm Diameter: 10 mm Filtration area: 0.11 m^2^ Pressure: 0.4 MPA	Turbidity: 99.2%	[119]
Physical	Sedimentation tank	HRT: 6 h Volume: 90 L	Turbidity: 18% TSS: 5.6% TVS: 27.5% BOD: 23.2%	[117]
Physical	Sedimentation tank	Rate: 2.49 m^3^/h	Turbidity: 27%	[118]
Biological	Anaerobic Sequencing Batch Reactor	HRT: 20 d Volume: 4 L	COD: 97% TSS: 96% TVS: 91%	[120]
Biological	Column photobioreactor	HRT: 7 d	TN: 90% TP: 90%	[121]
Biological	Tubular photobioreactor	Species: *Tetraslemis Suecica* HRT: 15 d Volume: 4 L	TN: 49% TP: 99%	[98]
Biological	Upflow anaerobic sludge blanket	Volume: 12 m^3^ Rate: 45 m^3^/h	Solid: 80%	[122]
Biological	Wetland	Plants: *Centella asiatica*, *Ipomoea aquatica*, *Salvinia molesta*, *Eichhornia crassipes*, and *Pistia stratiotes* HRT: 14 d Volume: 15 L	NH_3_-N: Max 98% TSS: Max 90% Phosphate: Max 64%	[123]
Biological	Wetland	Plants: *Ipomea asarifolia* HRT: 28 d	NH_3_-N: 85% TSS: 73% Phosphate: 53%	[124]
Biological	Wetland	Plants: *Azolla Pinnata* HRT: 14 d Volume: 10 L	NH_3_-N: 78% Phosphate: 79%	[125]
Physicochemical	Adsorption	Adsorbent: PAC Dosage: 3.5 g HRT: 45 min Mixing speed: 150 rpm	Turbidity: 91.4% TSS: 89.1%	[126]
Physicochemical	Advance oxidation process	Fenton oxidation	Antibiotics 89.9%	[127]
Physicochemical	Coagulation–flocculation	Compounds: PAC and polyamines Dosage: 32 mg/L Slow mixing: 5–15 min Settling: 15–60 min	Turbidity: 99.4% SS: 97.7% PO_4_-P: 98.2%	[128]
Physicochemical	Coagulation–flocculation	Compounds: Drewfloc Dosage: 5–10 mg/L Rapid mixing: 300 rpm 1 min Slow mixing: 20 rpm 10 min Settling: 15 min	TSS: 92% TP: 13.5% TN: 14.3%	[129]
Hybrid	Biofloc	HRT: 65 d Volume: 200 L	NH_4_^+^-N: 97%	[130]
Hybrid	Hybrid Moving Bed Biofilm Reactor	Volume: 8.8 L	TN: 43% TP: 84%	[131]
Hybrid	RAS		NO_3_–N: 80.88% PO_4_-P: 100%	[132]
Hybrid	RAS		NO_3_–N: 98.73% PO_4_-P: 99.46%	[133]
Hybrid	RAS		NO_3_–N: 40% PO_4_-P: 75%	[134]

BOD = biological oxygen demand, COD = chemical oxygen demand, TN = total nitrogen, NH_3_ = ammonia, NH_3_-N = ammonia nitrogen, NH_4_^+^ = ammonium, NH_4_-N = ammonium nitrogen, NO_3_^−^ = nitrate, NO_3_-N = nitrate nitrogen, TP = total phosphorus, TSS = total suspended solid, TVS = total volatile solid, PO_4_^3−^ = phosphate, PO_4_-P = orthophosphate.

To summarize the available options, a popular aquaculture system called RAS [135] utilizes physical treatment units, such as filtration (mostly membrane), for solid removal [136] and UV disinfection [137]. RAS is equipped with a biological treatment unit, such as a bioreactor (activated sludge) or a digestion system, to remove high organic and nutrient contents [120,138,139,140]. A biofloc system (also known as a “symbiotic process”), which can be used as an alternative to RAS, utilizes the interactions among organic matter, bacteria, and algae in a pond to produce pellets (or bioflocs) for fish feed [4,141]. Wetland is also a feasible option to remove nutrients from aquaculture wastewater before final release, because plants require nutrients for their growth while producing plant biomass [24,142]. In the physicochemical options, adsorption [143] and the advanced oxidation process (AOP) [144] can be used to produce clean effluents in an aquaculture system. Coagulation–flocculation is also considered one of the best practices in aquaculture treatment systems [145], in which solid/colloidal particles, nutrients, and organic matter are converted into sludge to be separated further. The produced sludge from this treatment has a high potential to be utilized further, rather than ending up in a landfill [63]. For instance, the sludge can be repurposed for bioenergy production, compost, or fertilizer, thereby promoting a circular economy in aquaculture wastewater management.

These treatment technologies should be integrated to obtain good-quality water for recycling. The integration approach not only ensures optimal performance of each unit, but also addresses site-specific challenges [146], such as seasonal variations in wastewater characteristics, space constraints, and cost limitations. To compare the available technologies for solid removal, filtration using sand removed 40% of TSS [147], and membrane filtration removed 94% of TSS [148]; adsorption using activated carbon removed 89% of TSS [126], whereas coagulation–flocculation using alum removed 94% of TSS [68]. In organic removal, membrane filtration can remove up to 76% BOD [148], aerobic bioreactor removed 56% of COD [149], moving bed biofilm reactor removed 80% of COD [150], anaerobic digester removed up to 97% of COD [151], 61% by wetland [152], 88% COD removed by activated carbon [153], 96% by advanced oxidation [154], and 75% COD removal by using coagulation–flocculation [145]. The autotrophic biofloc system also reduced the feeding requirement, which may lower the COD concentration, but its reduction was still unquantified [155]. In terms of nutrient removal, the moving bed biofilm reactor reached 92% of the TN [150] and 83% of TP removals [131], anaerobic digester removed 75% of TN and 98% of TP [151], whereas constructed wetlands removed >98% of TN and TP [152,156]. Coagulation–flocculation showed the best performance in removing solids, the bioreactor was considered better for organic removals, and wetland showed the best performance for nutrient removals. These findings underscore the importance of matching treatment methods with target pollutants, ensuring resource efficiency and cost-effectiveness. Depending on the initial characteristics of the aquaculture wastewater [157], the selection of the treatment integration needs to be carried out carefully to obtain good-quality water for recycling. A holistic evaluation, including economic feasibility, environmental impact, and operational complexity, is critical for designing sustainable aquaculture wastewater management systems.

## 5. Valorization of Aquaculture Wastewater

### 5.1. Utilization of Aquaculture Effluent into Aquaponic/Hydroponic

The use of aquaculture wastewater in aquaponic/hydroponic systems has been reported in previous works [27,158,159,160]. This initiative leads to simultaneous wastewater treatment and utilization of nutrient-rich effluents. Aquaculture effluents are rich in nutrients essential for plant growth, such as N, P, and K [12,158,161,162]. The nutrients in aquaculture also exist in a bioavailable state, making it an excellent growth medium for hydroponic plants [163]. A study reported a >75% uptake of nutrients (N, P, and K) from aquaculture wastewater using lettuce in an aquaponic system, simultaneously generating higher production than tap water feed [164]. A hydroponic system may also be used in the treatment of aquaculture wastewater via a biofiltration mechanism, apart from its nutrient uptake [165,166]. Utilization in agriculture was also assessed by Egbuikwem et al. [142] using edible crops of lettuce and beets, which reported that the utilization of aquaculture wastewater did not have a negative effect on seed germinations, although the residual recalcitrant must be considered because they reduced the chlorophyll pigment and decreased the root elongation compared with the control.

### 5.2. Utilization of Aquaculture Effluent as an Algae Cultivation Medium

Algae is known as one of the new paradigms of renewable energy because it can be valorized into various valuable products [167]. Insights into algae cultivation have shifted to the utilization of wastewater as a growth medium [168,169]. Aquaculture effluents are suitable options because they contain the required elements for algae production [170,171,172]. Guo et al. [147] reported the cultivation of *Platymonas subcordiformis* using aquaculture effluent with 8.9-fold higher algae density from the initial stage. Apart from biomass production, 95% and 99% N and P removal rates were achieved, respectively. Similarly, Hawrot-Paw et al. [148] also utilized aquaculture wastewater to grow *Chlorella minutissima* with productivity reaching 0.55 g/L.d. The used species was also able to remove N and P by up to 88% and 99%, respectively. Five species of microalgae, *Chlorella* sp., *Dunaliella* sp., *Nannochloropsis* sp., *Navicula* sp., and *Tetraselmis* sp., produced up to 188.5 mg/L yield with high fatty acid content during cultivation in a study using shrimp wastewater [102,173]. The cultivation of *Chlamydomonas reinhardtii* using aquaculture effluents showed the highest lipid content with the simultaneous removal of CO_2_ from the environment, making them a suitable candidate for CO_2_ sequestration [174].

### 5.3. Daphnid Cocultivation Using Aquaculture Effluent

Daphnids are one of the species used for fish feed, especially in the fry stage [55,175]. Daphnids can be grown in a nutrient-rich medium, which means aquaculture effluents may be used for this purpose [176,177]. Due to the minute size of the daphnids, they do not occupy a large space; thus, they can be cocultivated together with fish or algae. Cheban et al. [152] utilized aquaculture effluents to grow algae (*Desmodesmus armatus*) and *Daphnia magna* intermittently. Intermittent cultivation successfully resolved the daphnids’ continuous feeding (by algae existence), thereby producing daphnids with high lipid, protein, and carotenoid contents.

Rashid et al. [38] utilized aquaculture wastewater and aquaculture wastewater + yeast to assess the total daphnid production compared with tap water. Their results showed aquaculture wastewater + yeast produced 8.6-fold more individuals than tap water. The experiment involving 21 days of growth also revealed that aquaculture wastewater contained enough feed for the daphnids, whereas the one in tap water required periodic feeding. In another study, Azhar et al. [151] utilized flocs from tilapia pond culture as a supplement for *D. magna* cultivation compared with chicken manure and reported 5-fold higher individual growth.

### 5.4. Utilization of Aquaculture Sludge

As mentioned previously, aquaculture waste is known for its rich nutrient content [63,94]. Nutrient content in aquaculture mostly comes from excess fish feed and undigested feed, which are dissolved into water or deposited as the bottom sediment [76]. Digested feed excreted as feces also contains dissolved nutrients [178]. The high nutrient content in farming ponds can also induce the growth of algae, transforming the soluble nutrient into biomass [3]. The mentioned nutrient mostly consists of N and P [172]. N is mostly found in the form of ammonia, whereas nitrates and nitrites may also be found depending on the oxidation condition inside the pond [76]. Phosphate is the major constituent of P in aquaculture ponds [179]. The high concentrations of N and P are not only found in the water, but also in sludge [158,180] because the algae growth and the biological activities inside the pond may transform the soluble nutrients into solid form [76].

Considering the nutrient characteristics of aquaculture sludge, the potential of sludge utilization as soil fertilizer is attracting increasing interest [181]. Aquaculture–aquaponic is one of the integrated cultures that show promising sustainable applications [163,182,183]. Current studies on the use of aquaculture sludge as soil fertilizer are listed in Table 3.

**Table 3:** Research on aquaculture sludge as a soil fertilizer.

Culture	Sludge Characteristics	Conversion Method	Application System	Finding(s)	Reference
*Oreochromis niloticus* L.	C 35.5% N 3.87% P 31.27 mg/L K 28.1 mg/L	Anaerobic and aerobic digestion	Hydroponic	Aerobic treatment increased the soluble phosphorus by up to 3.2-fold and concentrated the K by up to 1.3-fold.	[183]
Salmon	C 229.6 g/kg N 82 g/kg NH_4_-N 6.9 g/kg NO_3_-N 0.027 g/kg P 24 g/kg K 8.2 g/kg	Drying and anaerobic digestion	Bioassay	Dried sludge achieved 50–80% of economic efficacy as compared to mineral fertilizer.	[184]
Atlantic salmon	N 74 g/kg P 54 g/kg	Raw and anaerobic digestion	Field study	Raw sludge can be used directly for land application. Consistent, good-quality biosolids can be obtained from digestion with odor elimination received with volatile solids below 60%. The concentrations of heavy metals were far below the permissible standard for land application.	[9]
Salmon	N 34.2 g/kg P 45.8 g/kg K 1.1 g/kg	Drying	Pot and field study	Optimization of P availability in fertilizer must be conducted because it showed low solubility in soil with low relative agroeconomic efficiency.	[11]
-	High P	Direct use	Field study	Aquaculture sludge not only functioned as soil fertilizer, but also supplied the whole P requirement for soybeans. A 50:50 ratio of sludge and soil was suggested as the optimum application.	[185]
*Litopenaeus vannamei*	C 10.82% N 0.7% P 1.59% K 0.9%	Composting	-	The utilization of indigenous bacteria isolated from the pond showed the same quality as the commercial activator. The N, P, and K contents are still below standard and suggested to be mixed with fish waste.	[12]
*-*	C 482.8 g/kg N 28.6 g/kg P 14.1 g/kg K 31.4 g/kg	Vermicomposting	-	The produced compost was suitable for agricultural applications. Protein-rich worm was produced as a byproduct suitable for fish feed.	[13]

The application of raw aquaculture sludge as a soil conditioner was reported by Asie et al. [161], whereas the utilization of raw aquaculture sludge as fertilizer was investigated by Madariaga and Marín [7]. Furthermore, the application of raw aquaculture sludge as fertilizer was proven feasible for improving crop yield, thus highlighting its potential for various agricultural purposes. One of the physical disadvantages of using raw aquaculture sludge is the unpleasant odor of the byproduct [9], but this can be solved through drying or composting treatment [186]. In addition to odor, raw sludge composition, such as residual chemicals and toxins, must be considered.

Drying is one of the simplest, but most feasible, treatments for aquaculture sludge prior to its utilization as a fertilizer [11,184]. Drying removes the water content from the sludge, leaving only the dry matter for direct land application [184]. However, due to the inconsistent quality of raw materials, drying may produce fertilizers of fluctuating quality. To resolve this issue, digestion and composting are proposed as a treatment to manufacture products with consistent quality [184].

Anaerobic digestion is one of the most economically feasible technologies to be used in further processing aquaculture sludge because it requires minimum energy input [9,183]. The digestate resulting from anaerobic digestion has the potential as fertilizer owing to the concentrates of the inputs [9]. By using anaerobic digestion, the byproducts of biogas can also be utilized further [170]. As an alternative to digestion, vermicomposting opens new opportunities for the treatment of aquaculture sludge, in which the byproducts of worm biomass may be used further as fish feed to achieve a circular economy in the aquaculture sector (discussed further in Section 6). The end product of vermicomposting meets the solid fertilizer standard, indicating its potential application in the agricultural sector [187,188,189].

## 6. Toward a Biocircular Economy in the Aquaculture Sector

**Figure 5:**
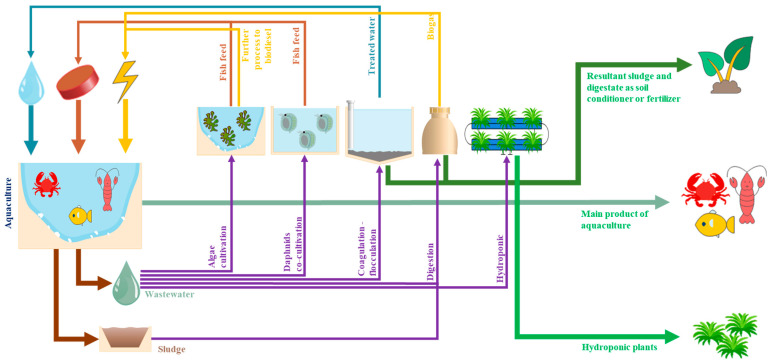
Adoption of the biocircular economy concept in the aquaculture sector.

A “circular economy” is defined as a closed loop of industry or sector by re-engaging the effluents/byproducts into the production cycle [190]. The term “biocircular economy” refers to the involvement of biomass valorization and/or the utilization of biotechnology to achieve a circular economy [191]. The aquaculture sector produces wastewater with rich nutrient content, and the resulting sludge also contains high organic matter with great economic value [192]. The previously mentioned items may be involved in achieving a biocircular economy in the aquaculture sector, as demonstrated in the scheme shown in Figure 5.

The main resources used in aquaculture ponds are water, fish pellets, and energy [76], and these inputs contribute to the production of main aquaculture products. Aquaculture production processes and the involvement of these raw materials generate byproducts, such as wastewater [193], sludge [158], and fish waste [194]. The biocircular economy in the aquaculture sector involves the processing of the generated biomass (in the form of sludge and fish wastes) and wastewater. Aquaculture wastewater has the characteristics of high organic and nutrient content, making it suitable to be utilized further to reclaim the valuable materials they contain [192]. As previously discussed, aquaculture wastewater contains high nutrients, and is very suitable as an algae cultivation medium [50,171,195]. Algae, in general, contains high lipid and carotenoid contents, which can be processed further into fish feed [195] and turned into renewable energy, such as biofuel [196,197]. The converted biomass produced using aquaculture wastewater may then be used as a substitute for energy utilization and as fish feed for subsequent aquaculture production.

The co-cultivation of daphnids is also highly feasible. Daphnids are organisms often used as fish feed [112]. Thus, the cultivation of daphnids may contribute to the substitution of fish feed materials, especially in the fry stage [169]. Despite being used as a growing medium, the treatment of aquaculture effluents is also mostly conducted using the coagulation–flocculation–sedimentation method [198]. The treated effluents can then be recycled back into the system, especially when nontoxic/nonmetal coagulants, such as natural coagulants/flocculants, are used during the process [63,199,200]. Additionally, when non-toxic coagulants are involved in the coagulation–flocculation–sedimentation process, the generated sludge, which is characterized by its high nutrients and minerals (Table 3), can further be used as biofertilizers. In turn, these fertilizers can become additional economical products outside of the production loop [178,201]. In addition, several researchers mentioned the integration of aquaculture and hydroponic systems to treat and reclaim nutrient-rich wastewater simultaneously for the cultivation of plants [24,27,159]. This approach is suitable for reducing nutrient content in aquaculture wastewater because plants uptake it as their nutrient source, further cultivating the biomass [183,202,203].

Similarly to wastewater, aquaculture sludge can undergo digestion. Slurry phase digestion can be performed by combining aquaculture wastewater and sludge for simultaneous processing [88]. Furthermore, to enhance the digestion efficiency, simultaneous digestion with fish waste can be conducted to maintain the C: N ratio [204]. As previously discussed in Section 4, anaerobic digestion can valorize the raw materials into biogas, which may be used further as additional energy [9,184]. The produced digestate from anaerobic digestion can also be utilized as soil fertilizer, which benefits many agricultural purposes [46,205,206]. An innovative way to process aquaculture sludge was proposed by Pounds [183], who wrote that a circular economy could be achieved by feeding aquaculture sludge to culture the larvae of black soldier flies. The nutrients and organic contents found in aquaculture sludge can be a good feed for the larvae [207]. In turn, such larvae can be harvested later as fish feed, closing the loop in the sludge treatment and feed supply.

## 7. Concluding Remarks and Future Research Directions

The biocircular economy is a game-changing technological development in aquaculture wastewater treatment because it represents improved sustainability in the environmental and economic aspects involved in the recovery and reuse of effluents. In particular, the presence of rich nutrients in aquaculture effluents provides a potential alternative to biofertilizers, especially because the conventional manufacturing of P-based fertilizers currently depletes P resources, whereas N-based fertilizers heighten carbon emissions. Furthermore, the valorization of aquaculture effluents and biomass for aquaponics/hydroponics, algae cultivation medium, cocultivation of daphnids, and biofertilizers has shown promising opportunities for the concurrent recovery of nutrients and the release of nontoxic wastewaters into external water bodies. With these examples, aquaculture wastewater treatment for effluent recovery could progressively change the economic model for wastewater management from a linear to a biocircular concept.

The transition towards a biocircular economy in the aquaculture sector also opens numerous opportunities for future research. The effort of wastewater treatment optimization can be focused on the integration of advanced techniques and multi-trophic aquaculture systems to maximize sludge dewatering, pathogen reduction, and nutrient retention. This effort can also lead to the innovative biofertilizer formulation for specific purposes. Future works are also suggested to explore a broader biocircular economy principle beyond waste valorization, which includes the exploration of synergetic relationships with other agricultural systems, industrial processes, and waste management. Understanding the barriers to technology adoption among aquaculture practitioners will benefit the adoption of the biocircular economy approach even further. Moreover, investigating the socio-economic impact of adopting biocircular economy practices in aquaculture, including the effort to conduct a cost–benefit analysis and the development of supportive policies, will encourage sustainable practices in aquaculture.

## Data Availability

The data that support the findings of this study are available from the corresponding author upon reasonable request.
